# Illness Severity in Hospitalized Influenza Patients by Virus Type and Subtype, Spain, 2010–2017

**DOI:** 10.3201/eid2602.181732

**Published:** 2020-02

**Authors:** Concepción Delgado-Sanz, Clara Mazagatos-Ateca, Jesús Oliva, Alin Gherasim, Amparo Larrauri

**Affiliations:** European Centre for Disease Prevention and Control, Stockholm, Sweden (C. Delgado-Sanz);; Institute of Health Carlos III, Madrid, Spain (C. Delgado-Sanz, C. Mazagatos-Ateca, J. Oliva, A. Gherasim, A. Larrauri);; CIBER Epidemiología y Salud Pública, Madrid (C. Delgado-Sanz, C. Mazagatos-Ateca, J. Oliva, A. Gherasim, A. Larrauri)

**Keywords:** influenza, surveillance, viral types, viral subtypes, severe influenza, influenza hospitalizations, influenza A(H1N1)pdm09, influenza A(H3N2), influenza B, viruses, Spain, respiratory infections

## Abstract

We conducted a retrospective cohort study to assess the effect of influenza virus type and subtype on disease severity among hospitalized influenza patients in Spain. We analyzed the cases of 8,985 laboratory-confirmed case-patients hospitalized for severe influenza by using data from a national surveillance system for the period 2010–2017. Hospitalized patients with influenza A(H1N1)pdm09 virus were significantly younger, more frequently had class III obesity, and had a higher risk for pneumonia or acute respiratory distress syndrome than patients infected with influenza A(H3N2) or B (p<0.05). Hospitalized patients with influenza A(H1N1)pdm09 also had a higher risk for intensive care unit admission, death, or both than patients with influenza A(H3N2) or B, independent of other factors. Determining the patterns of influenza-associated severity and how they might differ by virus type and subtype can help guide planning and implementation of adequate control and preventive measures during influenza epidemics.

## Abstract 2

Influenza A(H1N1)pdm09 caused more hospitalizations, intensive care unit admissions, and deaths than influenza A(H3N2) or B.

During the 2009 influenza pandemic, influenza surveillance activities were intensified in Spain (*1*). In accordance with international recommendations (*2*), Spain established surveillance of Severe Hospitalized Confirmed Influenza Case-patients (SHCIC) to monitor the evolution of severe influenza during pandemics and interpandemic influenza.

In the years since the 2009 influenza pandemic, SHCIC surveillance has become a consolidated severe influenza surveillance system that operates within the Spanish Influenza Surveillance System (*3*; Appendix). The system provides a standardized tool to monitor risk factors associated with severe influenza, identify influenza viruses associated with severe clinical manifestations, and monitor the disease burden of influenza epidemics. Sentinel hospitals belonging to the public health system of all 19 regions of Spain are involved in SHCIC surveillance (*3*).

The association of certain influenza virus types and subtypes with disease severity has been a major topic of influenza research in recent years (*4**–**10*). However, after the 2009 pandemic, findings on the severity of epidemics by type and subtype of influenza virus have varied widely. Some studies have reported no statistically significant differences in case-fatality rates and other markers of severity by type and subtype of influenza infections (*4*) and have shown that the risk for serious outcomes was not increased in hospitalized influenza patients infected with influenza A(H1N1)pdm09 (pH1N1) compared with seasonal influenza B viruses (*5*,*6*). In contrast, other authors have indicated that, in hospitalized influenza patients, pH1N1 infection is more clinically severe than influenza A(H3N2) or B infection (*7**–**10*).

SHCIC surveillance provides a reliable platform to monitor different influenza viruses associated with severe disease. By using the framework of this surveillance system, we aimed to explore disease severity of hospitalized influenza patients in Spain according to influenza virus type and subtype during the 7 influenza seasons that followed the 2009 pandemic.

## Material and Methods

We conducted a retrospective cohort study by using SHCIC surveillance data obtained across the 7 postpandemic influenza seasons (2010–11 through 2016–17). The SHCIC surveillance system is a passive, hospital-based surveillance system that includes 90–181 reporting hospitals during the study period; these hospitals are located throughout Spain and serve 45%–60% of the population of Spain, depending on the influenza season.

Each influenza season was defined as lasting from week 40 of the first year to week 20 of the following year. As part of the surveillance, clinicians in the participating hospitals were advised to swab any person with clinical suspicion of influenza-like illness (without specific case definition) and who required hospital admission to any hospital ward. A severe hospitalized confirmed influenza case-patient was defined as any person with a clinical profile compatible with influenza-like illness who had laboratory confirmation of influenza infection (Appendix) and who was hospitalized according to >1 of the following clinical criteria: pneumonia, acute respiratory distress syndrome (ARDS), multiple organ dysfunction syndrome (MODS), septic shock, or admission to an intensive care unit (ICU). The case-patient definition was unchanged throughout the study period.

Variables collected for surveillance purposes included demographic characteristics (age and sex), dates of symptom onset and hospitalization, virus type and subtype, presence of underlying medical conditions (any chronic respiratory, cardiovascular, renal or liver disease, class III obesity, diabetes mellitus, or immunosuppression), complications (pneumonia, any laboratory-confirmed viral or bacterial co-infection, ARDS, or MODS), antiviral treatment, time from symptom onset to start of antiviral treatment, influenza vaccination status, date of vaccination, admission to ICU, outcome (alive or dead), region, and influenza season. Class III obesity was defined as a body mass index >40 kg/m^2^. We obtained vaccination status by using clinical history and vaccination registries. We considered a patient to be correctly vaccinated if she or he received the vaccine >15 days before symptom onset.

We calculated the percentage of patients with a specific condition by using the number of patients with available information regarding the condition. Our analysis excluded patients whose influenza A subtype was unknown. We calculated the percentage of pregnant women by using all women of childbearing age (15–49 years of age) as the denominator.

We used univariate multinomial logistic regression models to compare demographic and clinical characteristics across virus types and subtypes, including as a dependent variable the influenza virus type and subtype, with pH1N1 used as reference, and as independent variables each of the characteristics of interest. We measured the effect of each predictor in the model as a relative risk ratio (RRR). We also conducted univariate logistic regression models to estimate the odds ratios (ORs) and 95% confidence intervals for the risk for clinical complications or death, considering influenza virus type and subtype to be an explanatory variable and using influenza pH1N1 as reference. We compared patients infected with influenza A(H3N2) or B against patients infected with pH1N1.

In addition, we applied multivariable logistic regression models, stratified by age group, to explore the effect of influenza virus type and subtype as an independent factor for the following severe outcomes: ICU admission, death, or both, using pH1N1 as reference. We adjusted all of these models for potential confounding such as sex, age, influenza season, underlying medical conditions, pneumonia, antiviral treatment, and receipt of seasonal trivalent influenza vaccine.

For all statistical analyses, we considered 2-sided p values <0.05 to be statistically significant. We performed the analyses by using Stata 14.0 (https://www.stata.com)

This study was conducted within the framework of ongoing SHCIC surveillance by the Institute of Health Carlos III National Epidemiology Centre. A formal ethics review was not required because the study was part of the routine surveillance activities in Spain. However, we collected anonymized data and obtained verbal consent from all patients before they were swabbed for surveillance purposes.

## Results

During September 2010–May 2017, a total of 12,942 case-patients were reported in Spain. We included 8,985 patients with complete influenza virus type and subtype information in our study; 4,568 (51%) were infected with pH1N1, 3,091 (34%) with influenza A(H3N2), and 1,326 (15%) with influenza B.

**Figure 1:**
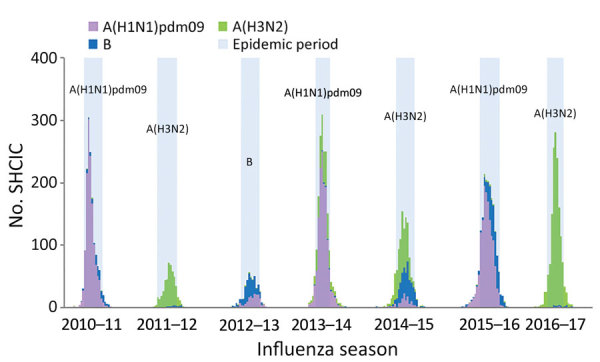
Number of patients hospitalized for laboratory-confirmed severe influenza, by influenza virus type or subtype and week of hospital admission, Spain, influenza seasons 2010–11 to 2016–17. Seasonal epidemic periods are labeled with dominant circulating virus.

SHCIC surveillance indicated week-by-week patterns that matched the epidemiologic patterns for influenza in the community based on the sentinel system for primary care. The identified influenza virus types and subtypes among case-patients were consistent with the type and subtype of influenza virus circulating within the general population (Figure 1). pH1N1 was the dominant subtype among case-patients during the 2010–11, 2013–14, and 2015–16 seasons; influenza A(H3N2) during the 2011–12, 2014–15, and 2016–17 seasons; and influenza B during the 2012–13 season. Also, we noted a substantial contribution from influenza B infections during the influenza A(H3N2)–dominant 2014–15 season and after the peak of the pH1N1-dominant 2015–16 influenza season (Figure 1). 

**Figure 2:**
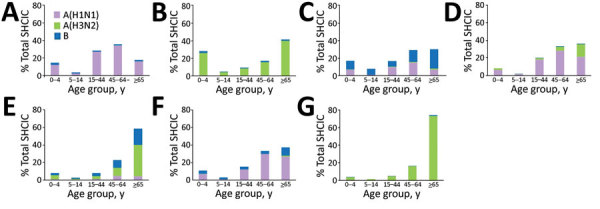
Number of patients hospitalized for laboratory-confirmed severe influenza, by influenza virus type or subtype and age group, Spain, influenza seasons 2010–11 to 2016–17. A) 2010–11 season. B) 2011–12 season. C) 2012–13 season. D) 2013–14 season. E) 2014–15 season. F) 2015–16 season. G) 2016–17 season.

The distribution of case-patients by age group varied according to the circulating virus type and subtype in each influenza season (Figure 2). In seasons with dominant pH1N1 circulation (Figure 2, panels A, C, and F), most patients (52%) were persons 15–64 years of age. Patients >65 years of age accounted for 65% of case-patients in those seasons with dominant influenza A(H3N2) circulation (Figure 2, panels B, E, and G); however, during the 2011–12 season, a relatively high percentage (33%) of case-patients were children. In the 2012–13 season, 54% of case-patients 15–64 years of age were infected with pH1N1, whereas 89% of case-patients 5–14 years of age were infected with influenza B (Figure 2, panel C). In general, case-patients infected with pH1N1 were significantly younger (median age 53 years [interquartile range (IQR) 37–66 years]) than those infected with influenza A(H3N2) (median age 73 years [IQR 56–83 years]) and influenza B (median age 60 years [22–74 years]) (p<0.001).

Regarding the clinical characteristics, case-patients infected with influenza A(H3N2) or B virus were more likely to have >1 underlying medical conditions compared with those with pH1N1 infection (crude RRR [cRRR] 2.81 [95% CI 2.45–3.22] for influenza A[H3N2]–infected patients and cRRR 1.32 [95% CI 1.13–1.55] for influenza B–infected patients) (Table 1). This pattern also was observed for chronic respiratory, cardiovascular, and renal diseases. However, immunosuppression was less likely among influenza A(H3N2)–infected patients than pH1N1-infected patients (cRRR 0.72 [95% CI 0.63–0.84]). Class III obesity was less frequent among influenza A(H3N2)–infected patients (cRRR 0.66 [95% CI 0.55–0.78]) and influenza B–infected patients (cRRR 0.59 [95% CI 0.46–0.75]) than among pH1N1-infected patients. Among women 15–49 years of age, fewer pregnancies were observed among influenza A(H3N2)–infected patients than pH1N1-infected patients (cRRR 0.33 [95% CI 0.17–0.64]). Patients infected with influenza A(H3N2) or B virus were less likely to receive antiviral treatment than those infected with pH1N1 (cRRR 0.48 [95% CI 0.42–0.54] for influenza A[H3N2]–infected patients and cRRR 0.30 [95% CI 0.26–0.34] for influenza B–virus infected patients) (Table 1). The median days from symptom onset to hospitalization was longer among pH1N1-infected patients (4 days [IQR 2–6 days]) than for influenza A(H3N2)–infected patients (3 days [IQR 1–5 days] (p<0.001), 

**Table 1:** Demographic and clinical characteristics of patients hospitalized for laboratory-confirmed severe influenza, by influenza virus type or subtype, Spain, influenza seasons 2010–11 to 2016–17*

Characteristic	Influenza virus type or subtype						
	pH1N1		A(H3N2)			B	
	No. (%)		No. (%)	Crude RRR† (95% CI)		No. (%)	Crude RRR‡ (95% CI)
Total no. patients	4,568 (100)		3,091 (100)	NA		1,326 (100)	NA
Age group, y							
<15	566 (12)		333 (11)	Referent		318 (24)	Referent
15–64	2,767 (61)		716 (23)	0.44 (0.38–0.52)		437 (33)	0.28 (0.24–0.33)
>65	1,231 (27)		2,035 (66)	2.81 (2.41–3.27)		567 (43)	0.82 (0.69–0.97)
Missing data	4 (0.1)		7 (0.2)	NA		4 (0.3)	NA
Sex							
M	2,589 (57)		1,637 (53)	0.86 (0.78–0.94)		736 (56)	0.96 (0.85–1.08)
F	1,977 (43)		1,453 (47)	Referent		588 (44)	Referent
Missing data	2 (0.1)		1 (0.1)	NA		2 (0.2)	NA
Underlying medical condition§	2,334 (68)		2,055 (86)	2.81 (2.45–3.22)		754 (74)	1.32 (1.13–1.55)
Missing data	1,161 (25)		700 (23)	NA		310 (23)	NA
Class III obesity (BMI >40 kg/m^2^)	447 (12)		205 (8)	0.66 (0.55–0.78)		80 (7)	0.59 (0.46–0.75)
Chronic respiratory diseases	686 (22)		680 (30)	1.53 (1.35–1.73)		238 (25)	1.20 (1.01–1.42)
Chronic cardiovascular diseases	800 (21)		1,051 (40)	2.49 (2.23–2.78)		308 (28)	1.45 (1.24–1.69)
Diabetes mellitus	696 (18)		755 (29)	1.81 (1.61–2.04)		225 (20)	1.15 (0.97–1.36)
Renal diseases	335 (9)		394 (15)	1.87 (1.60–2.18)		132 (12)	1.42 (1.14–1.75)
Chronic liver disease	212 (6)		147 (6)	1.02 (0.82–1.26)		58 (5)	0.94 (0.70–1.27)
Immunosuppression	632 (17)		327 (13)	0.72 (0.63–0.84)		170 (16)	0.92 (0.77–1.11)
Pregnancy¶	125 (24)		11 (10)	0.33 (0.17–0.64)		18 (24)	0.98 (0.56−1.73)
Missing data	134 (20)		30 (21)	NA		19 (20)	NA
Antiviral treatment	3,787 (86)		2,241 (75)	0.48 (0.42–0.54)		800 (64)	0.30 (0.26–0.34)
Missing data	165 (4)		86 (3)	NA		86 (6)	NA
Oseltamivir	3,709 (99.3)		2,209 (99.7)	NA		777 (99.4)	NA
Zanamivir	16 (0.4)		3 (0.1)	NA		4 (0.5)	NA
Other	11 (0.3)		5 (0.2)	NA		1 (0.1)	NA
Seasonal trivalent influenza vaccine	514 (14)		862 (36)	3.45 (3.05–3.91)		261 (27)	2.18 (1.84–2.58)
Missing data	961 (21)		727 (24)	NA		345 (26)	NA

*BMI, body mass index; RRR, relative risk ratio; NA, not applicable. †Influenza A(H3N2) compared with pH1N1 (reference).  ‡Influenza B compared with pH1N1 (reference).  §Underlying medical conditions defined as >1 of the following: class III obesity (BMI >40 kg/m2), chronic respiratory diseases, chronic cardiovascular diseases, diabetes mellitus, renal diseases, chronic liver disease, or immunosuppression. ¶Pregnancy among women of childbearing age (15–49 years).

The analysis of clinical complications and outcome revealed that patients with influenza A(H3N2) and B virus infection had lower risk for pneumonia (crude OR [cOR] 0.68 [95% CI 0.61–0.76] for influenza A[H3N2]–infected patients and cOR 0.77 [95% CI 0.67–0.89] for influenza B–virus infected patients), ARDS (cOR 0.69 [95% CI 0.61–0.77] for influenza A[H3N2]–infected patients and cOR 0.73 [95% CI 0.63–0.85] for influenza B–virus infected patients), and ICU admission (cOR 0.55 [95% CI 0.50–0.61] for influenza A[H3N2]–infected patients and cOR 0.64 [95% CI 0.56–0.73] for influenza B–virus infected patients) compared with patients with pH1N1 infection (Table 2). However, patients infected with influenza A(H3N2) or B had a higher risk for co-infection (cOR 1.23 [95% CI 1.09–1.38] for influenza A[H3N2]–infected patients and cOR 1.43 [95% CI 1.22–1.67] for influenza B–virus infected patients). The case-fatality rate was significantly higher among influenza A(H3N2)–infected patients (cOR 1.25 [95% CI 1.10–1.43]) than for pH1N1-infected patients. The risk for death was significantly lower for those patients infected with influenza B (cOR 0.76 [95% CI 0.62–0.93] for patients hospitalized and cOR 0.73 [95% CI 0.55–0.97] for those admitted to ICU) than patients with pH1N1 infection.

**Table 2:** Clinical complications and final outcomes of patients hospitalized for laboratory-confirmed severe influenza, by influenza virus type or subtype, Spain, influenza seasons 2010–11 to 2016–17*

Complication and outcome	Influenza virus type or subtype							
	pH1N1		A(H3N2)			B		
	No. (%)		No. (%)	Crude OR† (95% CI)		No. (%)	Crude OR‡ (95% CI)
Total no. patients	4,568 (100)		3,091 (100)	NA		1,326 (100)	NA
Pneumonia	3,529 (78)		2,154 (71)	0.68 (0.61–0.76)		951 (74)	0.77 (0.67–0.89)
Missing data	71 (2)		69 (2)	NA		36 (3)	NA
Co-infection	903 (26)		680 (31)	1.23 (1.09–1.38)		320 (34)	1.43 (1.22–1.67)
Missing data	1,153 (25)		873 (28)	NA		383 (29)	NA
ARDS	1,220 (29)		571 (22)	0.69 (0.61–0.77)		271 (23)	0.73 (0.63–0.85)
Missing data	405 (9)		528 (17)	NA		160 (12)	NA
MODS	405 (10)		236 (9)	0.94 (0.79–1.11)		107 (9)	0.94 (0.75–1.17)
Missing data	467 (10)		559 (18)	NA		177 (13)	NA
ICU admission	1,787 (41)		820 (28)	0.55 (0.50–0.61)		389 (31)	0.64 (0.56–0.73)
Missing data	245 (5)		146 (5)	NA		77 (6)	NA
Case-fatality rate							
Deaths in hospitalized patients	585 (14)		493 (16)	1.25 (1.10–1.43)		130 (11)	0.76 (0.62–0.93)
Deaths in ICU patients	405 (24)		180 (23)	0.90 (0.74–1.11)		68 (19)	0.73 (0.55–0.97)
Missing data	254 (6)		89 (3)	NA		109 (8)	NA

*ARDS, acute respiratory distress syndrome; ICU, intensive care unit; MODS, multiple organ dysfunction syndrome; NA, not applicable; OR, odds ratio. †OR of influenza A(H3N2) compared with pH1N1 (reference).  ‡OR of influenza B compared with pH1N1 (reference).

We used a multivariable logistic regression analysis to explore the effect of influenza virus type and subtype on the severity of outcomes, such as ICU admission, death, or ICU admission and death, according to age group (Appendix Table 1). Case-patients >15 years of age who had influenza A(H3N2) or B infection showed less risk for death or ICU admission than patients infected with pH1N1, independent of other risk factors (Appendix Table 1). The pattern for all case-patient age groups combined was similar. When we compared pH1N1-infected patients with the other 2 patient groups, we observed significant differences in risk for ICU admission among influenza A(H3N2)–infected patients (adjusted OR [aOR] 0.56 [95% CI 0.44–0.71]) and influenza B–infected patients (aOR 0.51 [95% CI 0.41–0.63]); risk for death among influenza A(H3N2)–infected patients (aOR 0.56 [95% CI 0.40–0.77]) and influenza B–infected patients (aOR 0.38 [95% CI 0.26–0.54]); and risk for ICU admission and death among influenza A(H3N2)–infected patients (aOR 0.59 [95% CI 0.47–0.73]) and for influenza B–infected patients (aOR 0.50 [95% CI 0.44–0.62]). However, among children <15 years of age, we observed no significant differences in the severity of outcome by virus type and subtype. In addition, we observed no difference in the risk for ICU admission between different influenza A subtypes among patients >65 years of age (Appendix Table 1).

## Discussion

Our findings show that SHCIC surveillance has provided valuable information on disease severity by influenza virus type and subtype since the 2009 pandemic. We found that case-patients infected with pH1N1 were significantly younger than those infected with influenza A(H3N2) or B and had a higher risk for clinical complications and severe outcomes, such as ICU admission, death, or both compared with case-patients with influenza A(H3N2) or B virus infections.

During the 2011–12 and 2012–13 seasons, an unexpectedly low number of case-patients were reported compared with previous seasons. Similar observations were reported during the 2011–12 influenza season in the United States (*11*) and France (*8*), where influenza A(H3N2) was also the predominant virus and caused excess mortality in the elderly (*12**–**14*). Given that the definition of case-patient was established in a season with almost exclusively pH1N1 circulation, the figures for the first postpandemic season with dominant influenza A(H3N2) virus might have been affected by lower definition sensitivity for identifying case-patients infected with other influenza types and subtypes. In addition, according to 2 international cohort studies conducted during 2009–2015 (*15*), outpatients with influenza A(H3N2) virus infection were less likely to be hospitalized than those infected with pH1N1 or influenza B virus, which might have influenced the numbers reported. Another aspect that could influence the higher number of pH1N1 infections recorded compared with other subtypes is the wider availability of the PCR assay for this virus subtype since the 2009 pandemic in all the laboratories of the hospitals participating in SHCIC surveillance. 

Our results are similar to those from previous studies, which found that hospitalized influenza pH1N1-infected patients were younger than those infected with influenza A(H3N2) or B (*15*). Also, in the United States, a higher proportion of pH1N1 infections occurred in adults 15–64 years of age compared with influenza A(H3N2) and B infections (*7*). Several observations could be consistent with the differences on age by influenza virus type and subtype found in this study and others. The different susceptibility of each birth cohort is based on the likelihood that their influenza primary infections were with group 1 or 2 hemagglutinin. Individuals born before 1956 likely had their first infection with a group 1 virus and had preexisting cross-reactive antibodies against viruses of the same group as pH1N1 virus, whereas those born in 1968 or later appear protected against severe influenza A(H3N2) infection (*16**–**18*). Moreover, seasonal influenza A(H1N1) virus that circulated before 2009 provided some additional cross-reactive immunity protection in older patients against the newer pH1N1 virus (*17*,*18*). The younger patients, who have less exposure to this older seasonal influenza A(H1N1) virus, might have experienced more severe disease as a result of direct infection by pH1N1 and the resulting cytokine-induced inflammatory responses, an immune-mediated pathologic process which is believed to play an important role in the onset of severe disease (*19**–**21*).

In our study, case-patients infected with influenza A(H3N2) or B viruses were more likely to have underlying medical conditions than those infected with pH1N1. This observation is partly in line with findings from the aforementioned international cohort study (*15*) and could be consistent with the age difference between influenza virus type and subtype. However, when we stratified the analysis by age, the differences between those pH1N1-infected patients compared with influenza A(H3N2) and B remained significant, regardless of age (Appendix Tables 2–4). In contrast, a study in Argentina reported that the prevalence of underlying medical conditions did not differ between hospitalized patients with influenza A(H3N2) or pH1N1 infection (*10*).

We found that morbid obesity was more common among case-patients infected with pH1N1. This result accords with a higher prevalence of obesity (18.2%) found by another study in hospitalized patients with pH1N1 infection compared with patients with influenza A(H3N2) or B infection (<10%) (*7*). Obesity was first identified as a novel independent risk factor for influenza severity in hospitalized adults during the 2009 pandemic in California (USA) (*22*). Furthermore, another study found a stronger association between obesity and ICU admission and death for pH1N1 infections (*23*).

Our results indicate that the likelihood of pneumonia was higher among patients with pH1N1 than patients with influenza A(H3N2) or B infections. However, patients with influenza A(H3N2) or B infections had a higher risk for bacterial or viral co-infection. Although our study lacks information on other clinical features or radiologic findings, the results seem to be in line with previous studies. A US study found that adults with pH1N1 infection had an increased risk for radiographically confirmed pneumonia compared with those with influenza A(H3N2) infection (*24*). A study in Japan showed that hospitalized patients with pH1N1 virus had primary viral pneumonia more frequently and had mixed bacterial or secondary bacterial pneumonia less frequently compared with patients with influenza A(H3N2) or B virus infections (*25*). Another study, conducted during the first postpandemic influenza season, showed that patients with pH1N1 pneumonia had similar clinical characteristics but slightly higher disease severity and stronger systemic inflammatory response than patients with influenza A(H3N2) pneumonia (*26*). In addition, in our study, ARDS occurred more frequently in patients infected with pH1N1 than those infected with influenza A(H3N2) or B viruses, which accords with previous reports from other countries (*7*,*8*)

Treatment with antiviral drugs was significantly less common in patients with influenza A(H3N2) or B infections than in patients with pH1N1 infection, regardless of age (Appendix Tables 2–4). We were unable to explain these data because antiviral treatment is recommended for everyone hospitalized with influenza in Spain (*27*), and the virus type and subtype should not have influenced treatment decisions (*28*).

Our results indicate that patients with influenza A(H3N2) or B infections were less likely to be admitted to an ICU, die, or both than were those with pH1N1 infections, after controlling for potential confounders. These findings are in agreement with other studies of disease severity by influenza virus type and subtype, which report higher ORs for ICU admission for pH1N1-infected patients (*7*,*9*,*10*,*29*). The aforementioned international cohort study showed similar results to our own study for every age group except persons >65 years of age, for which they found higher hospitalization rates for outpatients infected with influenza B (*15*). In contrast, a study in South Africa showed no association between virus type and subtype and ICU admission or death (*4*). Other studies did not find differences in patient mortality between influenza A virus subtypes (*10*), or between other types or subtypes (*8*,*25*). In our study, we did not find differences in the risk for ICU admission by influenza A subtype in patients <65 years of age.

We should distinguish at this point the clinical seriousness caused by different influenza virus types and subtypes, as observed in severe influenza surveillance systems, from those results on the effect of influenza on population mortality rates obtained from population-based studies that use regression models. As previously reported, influenza A(H3N2)–dominant epidemics have a considerable impact on mortality, with highest excess mortality attributable to influenza occurring mainly in older adults (*12**–**14*,*30*,*31*). In addition, a study suggests that influenza B might also be more of a concern in terms of excess mortality in the influenza season 2017–18 (*32*). However, many of these deaths might have occurred in older persons who have a cascade of illness after an influenza infection, and influenza in older patients might not have a typical clinical profile. Moreover, many older patients might die at home or in managed care facilities and might not get to a hospital. The increase in deaths associated with influenza A(H3N2) at the population level might reflect greater population susceptibility or reduced vaccine effectiveness against influenza A(H3N2) that has become apparent in recent years (*33*), although it might not reflect the relative clinical seriousness of the individual infection. Therefore, our finding that pH1N1 infections caused a higher clinical seriousness in hospitalized patients than influenza A(H3N2) or B infections is fully congruent with the greater effect on the population mortality caused by influenza A(H3N2) seasons (*12**–**14*,*30*,*31*).

This study has several limitations. First, we cannot exclude a possible bias that results from using hospitalized case-based surveillance systems with many reporting sites that might have had different testing practices and might also have varied by season. However, because of the high percentage of the national population included in the SHCIC surveillance, the results obtained should be highly representative of the entire country. During the last 4 seasons, a relatively high proportion of influenza A infections were not subtyped, probably because of the implementation of rapid tests for influenza confirmation. Influenza testing could also have been biased depending on age, severity of symptoms, changes in swabbing practices in the last few seasons, or even as a result of the selection of patients for swabbing based on physician-suspected influenza; however, these factors should not have influenced the virus type and subtype recorded. The multivariable analysis has been controlled for bias by season to avoid potential biases related to the inclusion of several seasons in the study (i.e., differing dominant influenza virus types and subtypes and their antigenic drifts and shifts, influenza vaccine uptake, and seasonal variations in match the vaccine to the circulating influenza strains could all complicate comparisons between seasons). However, a real strength of this study is its representativeness; it enrolled patients from hospitals throughout Spain and across every age group, it covered every influenza season since SHCIC surveillance began in 2009, and it benefited from substantial virus co-circulation and a large sample size.

In conclusion, our findings suggest that hospitalized patients infected with pH1N1 virus had a higher risk for ICU admission, death, or both than patients infected with influenza A(H3N2) or B infections, despite being younger and having fewer underlying medical conditions. Therefore, in those seasons with considerable circulation of pH1N1, more admissions to hospital ICUs should be expected, especially among hospitalized young adult patients. To decrease treatment delays, antiviral treatment should be started shortly after admission to hospital when influenza is suspected. These observations could be of crucial importance when planning resource deployment during influenza epidemics. Understanding the patterns of disease severity associated with influenza and how these patterns might differ among virus types and subtypes can help guide public health measures to control influenza. This knowledge can help in directing resource allocation in the healthcare system during each influenza season and thus can ensure an effective response to pressures on ICUs, especially during pH1N1 epidemics.

Appendix Additional information about illness severity in hospitalized influenza patients, by virus type and subtype, Spain, 2010–2017.
