# Supplementary material for: Illness Severity in Hospitalized Influenza Patients by Virus Type and Subtype, Spain, 2010–2017
Source: Emerg Infect Dis. 2020 Feb;26(2):220–8. doi: 10.3201/eid2602.181732 (PMC6986827; doi:10.3201/eid2602.181732)
Supplement: Appendix — Additional information about illness severity in hospitalized influenza patients, by virus type and subtype, Spain, 2010–2017. [file 18-1732-Techapp-s1.pdf]

Article DOI: DOI: https://doi.org/10.3201/eid2602.181732

Illness Severity in Hospitalized Influenza Patients by Virus Type and Subtype, Spain, 2010–2017

Appendix

Severe Hospitalized confirmed Influenza Cases-Patients Surveillance System

The Severe Hospitalized confirmed Influenza cases-patients (SHCIC) Surveillance System was initiated during the 2009 influenza pandemic and has been consolidated throughout the post-pandemic seasons (2010–11/2017–18). In this surveillance system a network of hospitals and public health units at regional level located throughout Spain are participating. The Surveillance System, which operates within the Spanish Influenza Surveillance System, is coordinated by National Center of Epidemiology (CNE), Carlos III Health Institute (ISCIII).

Main Surveillance Objectives

• To describe the epidemiologic and virological characteristics of severe influenza disease

• To identify risk factors for severe influenza disease by age group

• To describe the burden of severe influenza disease by age and risk groups

• To compare the pattern of severity among influenza epidemics or pandemics

• To provide information to public health policy makers to guide the influenza prevention and control measures

Case-Patient Definition

Any person who present a clinical picture compatible with influenza and who require hospital admission will be swabbed. From those, a severe hospitalized confirmed influenza case-patient is any person who is hospitalize with at least one of the following clinical criteria:

pneumonia, acute respiratory distress syndrome (ARDS), multiple organ dysfunction syndrome (MODS), septic shock or admission to intensive care unit (ICU), AND is laboratory confirmed influenza. Laboratory confirmation is defined as a positive result from viral culture, reverse transcription-PCR (RT-PCR), identification of influenza virus antigen or antibody testing.

Population under Surveillance and Geographic Coverage

In the surveillance of SHCIC, hospitals designated for that purpose by each of the 19 Autonomous Regions are involved, ranging from 181, in the 2010–2011 season, to 90 in the 2013–2014 season. The population under surveillance correspond to the reference catchment population of these hospitals ranging from 59% in the 2010–2011 season to 40% in the 2012–2013 season. In the 2017–18 season, the population included in the surveillance of SHCIC was 23,460,172 inhabitants, which represents 51% of the Spanish population.

Type of Surveillance

SHCIC surveillance system is a hospital-based surveillance considered passive, voluntary and prospective.

Specification of Data Collection

- Case-based dataset format
- Key variables:
    - Demographic: age, sex, region
    - Dates of hospital admission, symptom onset, influenza vaccination, antiviral treatment and hospital discharge
    - Virus type and subtype
    - Genetically characterized influenza virus
    - Clinical: Underlying medical conditions and complications
    - Antiviral treatment and influenza vaccination status

▪ Outcome: ICU admission, death

- Frequency of data collection: weekly

- Responsible for data collection: Heath professionals from Preventive Medicine Departments at the hospitals involved in SHCIC surveillance system, and Public Health professionals at regional level responsible for such surveillance.

Flowchart of Spanish Severe Hospitalized Confirmed Influenza Case-Patients Surveillance System

Appendix Figure shows the steps included in the case collection, data flow, data analysis and dissemination of results, which are within the SHCIC surveillance system

Reporting Format

The data are collect on different electronic format at regional level, and subsequently they are transformed in a standard txt file

Data Entry

Weekly, each regional surveillance network is responsible for entering the data through a Web application of the Spanish Influenza Surveillance System (http://vgripe.isciii.es). Data are weekly retrieved at the National Center of Epidemiology, which is responsible for their analysis and the elaboration and release of the National Weekly Influenza Report.

Statistical Models

Univariate multinomial logistic regression models were performed to compare demographic and clinical characteristics across influenza virus types and subtypes. Including as dependent variable the influenza virus type and subtype, considering as reference category A(H1N1)pdm09 (pH1N1); and as independent variables each of the characteristics of interest: age groups (0–14, 15–64, <64 years), sex, presence of underlying medical conditions (chronic respiratory, cardiovascular, renal or liver diseases, class III obesity, diabetes mellitus, or

immunosuppression), antiviral treatment and seasonal influenza vaccination status. The effect of each predictor in the model is measured as relative risk ratio (RRR).

The regression equation used in univariate analyses for each variable of interest:

Log (P(virus = AH3N2)/P(virus = pH1N1)) = b10+b11*variable + Ɛ

Log (P(virus = B)/P(virus = pH1N1)) = b20+b21*variable + Ɛ

In addition, we performed the same analysis stratified by age groups (0–14, 15–64, <64 years) to investigate what the effect of age is on some of the associations found. The results of these analyzes are shown in Appendix Tables 2–4.

To estimate the odds ratios (OR) for developing clinical complications or death, we also conducted univariate logistic regression models, considering as dependent variables in each of the models, each of possible complications: pneumonia, any laboratory-confirmed viral or bacterial co-infection, acute respiratory distress syndrome (ARDS), multiple organ dysfunction syndrome (MODS) or admission to an intensive care unit (ICU); and death. Explanatory variable studied was the influenza virus type and subtype, taking as reference category pH1N1 virus. We compared patients infected with A(H3N2) or B against patients with pH1N1.

The regression equations used:

Log (odds of pneumonia) = α + β1*virus + Ɛ

Log (odds of co-infection) = α + β1*virus + Ɛ

Log (odds of ARDS) = α + β1*virus + Ɛ

Log (odds of MODS) = α + β1*virus + Ɛ

Log (odds of ICU) = α + β1*virus + Ɛ

Log (odds of death) = α + β1*virus + Ɛ

Multivariate logistic regressions models were performed to explore the effect of influenza virus type and subtype as an independent factor for the following severe outcomes: 1) admission to ICU, 2) death and 3) either admission to ICU or death, or both, using pH1N1 as the reference category. All these models were adjusted for potential confounding: sex, age, influenza season, underlying medical conditions (comorbidity), pneumonia, antiviral treatment and seasonal

trivalent influenza vaccine. We also performed the same analysis stratified by age groups (0–14, 15–64, <64 years). The results of these analyses are shown in Appendix Table 1.

The regression equations used in adjusted analyses are:

$$\text{Log (odds of ICU)} = \alpha + \beta 1 * \text{virus} + \beta 2 * \text{sex} + \beta 3 * \text{age} + \beta 4 * \text{season} + \beta 5 * \text{comorbidity} + \beta 6 * \text{pneumonia} + \beta 6 * \text{antiviral} + \beta 8 * \text{vaccine} + \varepsilon$$

$$\text{Log (odds of death)} = \alpha + \beta 1 * \text{virus} + \beta 2 * \text{sex} + \beta 3 * \text{age} + \beta 4 * \text{season} + \beta 5 * \text{comorbidity} + \beta 6 * \text{pneumonia} + \beta 6 * \text{antiviral} + \beta 8 * \text{vaccine} + \varepsilon$$

$$\text{Log (odds of ICU or death)} = \alpha + \beta 1 * \text{virus} + \beta 2 * \text{sex} + \beta 3 * \text{age} + \beta 4 * \text{season} + \beta 5 * \text{comorbidity} + \beta 6 * \text{pneumonia} + \beta 6 * \text{antiviral} + \beta 8 * \text{vaccine} + \varepsilon$$

Appendix Table 1. Effect of Influenza virus type or subtype on the severity outcome of laboratory-confirmed patients hospitalized for severe influenza, by age group, Spain, influenza seasons 2010–11 to 2016–17*

Age group and influenza virus type or subtype	ICU†			Death‡			ICU/ death§		
	No. (%)	Crude OR (95% CI)	Adjusted OR (95% CI)	No. (%)	Crude OR (95% CI)	Adjusted OR (95% CI)	No. (%)	Crude OR (95% CI)	Adjusted OR (95% CI)
0–14 y									
pH1N1	144 (27)	Ref	Ref	10 (2)	Ref	Ref	145 (28)	Ref	Ref
A(H3N2)	95 (29)	1.10 (0.81–1.50)	0.64 (0.31–1.12)	3 (1)	0.50 (0.14–1.83)	0.15 (0.01–2.77)	96 (30)	1.10 (0.81–1.50)	0.63 (0.30–1.30)
B	80 (26)	0.94 (0.69–1.30)	0.69 (0.41–1.15)	4 (1)	0.74 (0.23–2.38)	0.12 (0.01–1.29)	81 (28)	1.01 (0.73–1.39)	0.70 (0.42–1.17)
15–64 y									
pH1N1	1,239 (47)	Ref	Ref	318 (12)	Ref	Ref	1,304 (51)	Ref	Ref
A(H3N2)	290 (43)	0.83 (0.70–0.99)	0.68 (0.46–0.99)	76 (11)	0.88 (0.67–1.15)	0.51 (0.27–0.97)	316 (47)	0.86 (0.72–1.02)	0.61 (0.41–0.90)
B	160 (39)	0.71 (0.58–0.88)	0.59 (0.42–0.84)	29 (7)	0.57 (0.38–0.84)	0.32 (0.16–0.63)	169 (44)	0.75 (0.60–0.93)	0.55 (0.38–0.77)
>64 y									
pH1N1	402 (34)	Ref	Ref	256 (22)	Ref	Ref	516 (45)	Ref	Ref
A(H3N2)	434 (22)	0.56 (0.47–0.65)	0.79 (0.55–1.14)	410 (21)	0.94 (0.79–1.12)	0.59 (0.39–0.87)	717 (38)	0.75 (0.65–0.87)	0.70 (0.51–0.97)
B	149 (28)	0.76 (0.61–0.95)	0.56 (0.38–0.84)	96 (18)	0.80 (0.62–1.04)	0.46 (0.29–0.73)	200 (39)	0.81 (0.65–0.99)	0.53 (0.37–0.75)
All ages									
pH1N1	1,787 (41)	Ref	Ref	585 (14)	Ref	Ref	1,967 (46)	Ref	Ref
A(H3N2)	820 (28)	0.55 (0.50–0.61)	0.56 (0.44–0.71)	493 (16)	1.25 (1.10–1.43)	0.56 (0.40–0.77)	1,133 (39)	0.74 (0.67–0.81)	0.59 (0.47–0.73)
B	389 (31)	0.64 (0.56–0.73)	0.51 (0.41–0.63)	130 (11)	0.76 (0.6–0.93)	0.38 (0.26–0.54)	451 (38)	0.71 (0.62–0.81)	0.50 (0.40–0.62)

*Multivariate analysis adjusted for sex, age, season influenza, underlying medical conditions, pneumonia, antiviral treatment, and seasonal trivalent influenza vaccine. ICU, intensive care unit; OR, odds ratio.
†ICU case-patients compared with non–ICU case-patients.
‡Death case-patients compared with non–death case-patients.
§Either ICU admission or death, or both case-patients compared with non–ICU and non–death case-patients.

Appendix Table 2. Demographic and clinical characteristics of severe hospitalized confirmed influenza case-patients <15 y old, by type and subtype of influenza virus and age group in Spain, seasons 2010–11 to 2016–17*

	Type and subtype of influenza virus				
	pH1N1	A(H3N2)		B	
	n = 566	n = 333	Crude RRR†	n = 318	Crude RRR‡
Characteristic	No. (%)	No. (%)	(95% CI)	No. (%)	(95% CI)
Sex (males)	320 (56)	201 (60)	1.17 (0.89–1.54)	164 (52)	0.82 (0.62–1.08)
Underlying medical condition§	107 (26)	88 (39)	1.86 (1.32–2.63)	98 (40)	1.96 (1.40–2.74)
Class III obesity (BMI ≥40 kg/m2)	3 (0.6)	1 (0.3)	0.56 (0.06–5.42)	1 (0.4)	0.62 (0.06–6.02)
Chronic respiratory diseases	37 (9)	26 (9)	1.07 (0.63–1.81)	25 (10)	1.20 (0.70–2.04)
Chronic cardiovascular diseases	22 (4)	13 (4)	0.99 (0.49–1.99)	27(10)	2.42 (1.35–4.34)
Diabetes mellitus	7 (1)	6 (2)	1.45 (0.48–4.37)	11 (4)	2.99 (1.15–7.83)
Renal diseases	6 (1)	1 (0.3)	0.28 (0.03–2.38)	6 (2)	1.93 (0.62–6.05)
Chronic liver disease	2 (0.4)	1 (0.3)	0.84 (0.76–9.31)	1 (0.4)	0.94 (0.08–10.38)
Immunosuppression	18 (4)	13 (4)	1.23 (0.60–2.55)	13 (5)	1.37 (0.66–2.83)
Antiviral treatment	293 (56)	124 (39)	0.51 (0.38–0.68)	118 (41)	0.56 (0.42–0.75)
Seasonal trivalent influenza vaccine	16 (3)	24 (8)	2.62 (1.37–5.01)	19 (8)	2.46 (1.24–4.87)

*RRR, relative risk ratio; CI, confidence interval; BMI, body mass index.
†A(H3) vs. pH1N1 (reference). ‡B vs. pH1N1 (reference).
§Underlying medical conditions: one or more of class III obesity, (BMI ≥40 kg/m2), chronic respiratory diseases, chronic cardiovascular diseases, diabetes mellitus, renal diseases, chronic liver disease, or immunosuppression.

Appendix Table 3. Demographic and clinical characteristics of severe hospitalized confirmed influenza cases-patients between 15–64 y old, by type and subtype of influenza virus and age group in Spain, seasons 2010–11 to 2016–17*

	Type and subtype of influenza virus				
	pH1N1	A(H3N2)		B	
	n = 2,767	n = 716	Crude RRR†	n = 437	Crude RRR‡
Characteristic	No. (%)	No. (%)	(95% CI)	No. (%)	(95% CI)
Sex (males)	1,542 (56)	378 (53)	0.89 (0.75–1.05)	250 (57)	1.07 (0.87–1.31)
Underlying medical condition§	1,386 (67)	439 (84)	2.50 (1.94–3.21)	239 (74)	1.39 (1.07–1.81)
Class III obesity (BMI ≥40 kg/m2)	330 (15)	63 (11)	0.72 (0.54–0.96)	38 (11)	0.71 (0.50–1.01)
Chronic respiratory diseases	366 (19)	151 (30)	1.74 (1.39–2.17)	73 (24)	1.31 (0.99–1.75)
Chronic cardiovascular diseases	335 (14)	107 (18)	1.29 (1.01–1.62)	55 (15)	0.97 (0.71–1.31)
Diabetes mellitus	375 (16)	130 (21)	1.42 (1.14–1.78)	56 (16)	1.15 (0.97–1.36)
Renal diseases	141 (6)	51 (8)	1.44 (1.03–2.01)	30 (8)	1.41 (0.93–2.12)
Chronic liver disease	168 (7)	72 (12)	1.74 (1.30–2.31)	33 (9)	1.31 (0.88–1.93)
Immunosuppression	414 (18)	133 (22)	1.31 (1.05–1.63)	85 (24)	1.44 (1.10–1.88)
Antiviral treatment	2,457 (91)	586 (84)	0.48 (0.38–0.62)	310 (74)	0.27 (0.21–0.35)
Seasonal trivalent influenza vaccine	220 (10)	116 (19)	2.20 (1.72–2.81)	57 (19)	1.85 (1.35–2.54)

*RRR, relative risk ratio; CI, confidence interval; BMI, body mass index.
†A(H3) vs. pH1N1 (reference).
‡B vs. pH1N1 (reference).
§Underlying medical conditions: one or more of class III obesity, (BMI ≥40 kg/m2), chronic respiratory diseases, chronic cardiovascular diseases, diabetes mellitus, renal diseases, chronic liver disease, or immunosuppression.

Appendix Table 4. Demographic and clinical characteristics of severe hospitalized confirmed influenza cases-patients between >64 y of age, by type and subtype of influenza virus and age group in Spain, seasons 2010–11 to 2016–17*

	Type and subtype of influenza virus				
	pH1N1	A(H3N2)		B	
	n = 1,231	n = 2,035	Crude RRR†	n = 567	Crude RRR‡
Characteristic	No. (%)	No. (%)	(95% CI)	No. (%)	(95% CI)
Sex (males)	725 (59)	1,057 (52)	0.76 (0.65–0.87)	321 (57)	0.91 (0.75–1.12)
Underlying medical condition§	838 (90)	1,523 (93)	1.48 (1.11–1.97)	413 (92)	1.35 (0.89–2.03)
Class III obesity (BMI ≥40 kg/m2)	113 (12)	140 (9)	0.70 (0.54–0.90)	41 (9)	0.72 (0.50–1.05)
Chronic respiratory diseases	283 (35)	502 (34)	0.97 (0.81–1.16)	140 (36)	1.03 (0.80–1.33)
Chronic cardiovascular diseases	443 (45)	928 (54)	1.40 (1.20–1.64)	222 (47)	1.08 (0.86–1.34)
Diabetes mellitus	313 (32)	617 (36)	1.20 (1.02–1.42)	157 (34)	1.15 (0.97–1.36)
Renal diseases	188 (19)	341 (20)	1.06 (0.87–1.29)	94 (20)	1.05 (0.80–1.39)
Chronic liver disease	42 (4)	74 (4)	1.01 (0.69–1.49)	24 (5)	1.20 (0.72–2.00)
Immunosuppression	200 (21)	180 (11)	0.46 (0373–0.57)	72 (16)	0.70 (0.52–0.95)
Antiviral treatment	1,033 (87)	1,526 (77)	0.50 (0.41–0.61)	370 (69)	0.34 (0.26–0.43)
Seasonal trivalent influenza vaccine	447 (42)	1,086 (59)	1.99 (1.71–2.33)	249 (55)	1.64 (1.32–2.05)

*RRR, relative risk ratio; CI, confidence interval; BMI, body mass index.
†A(H3) vs. pH1N1 (reference).
‡B vs. pH1N1 (reference).
§Underlying medical conditions: one or more of class III obesity, (BMI ≥40 kg/m2), chronic respiratory diseases, chronic cardiovascular diseases, diabetes mellitus, renal diseases, chronic liver disease, or immunosuppression.
¶ Pregnancy among women of childbearing age (15–49 y of age).

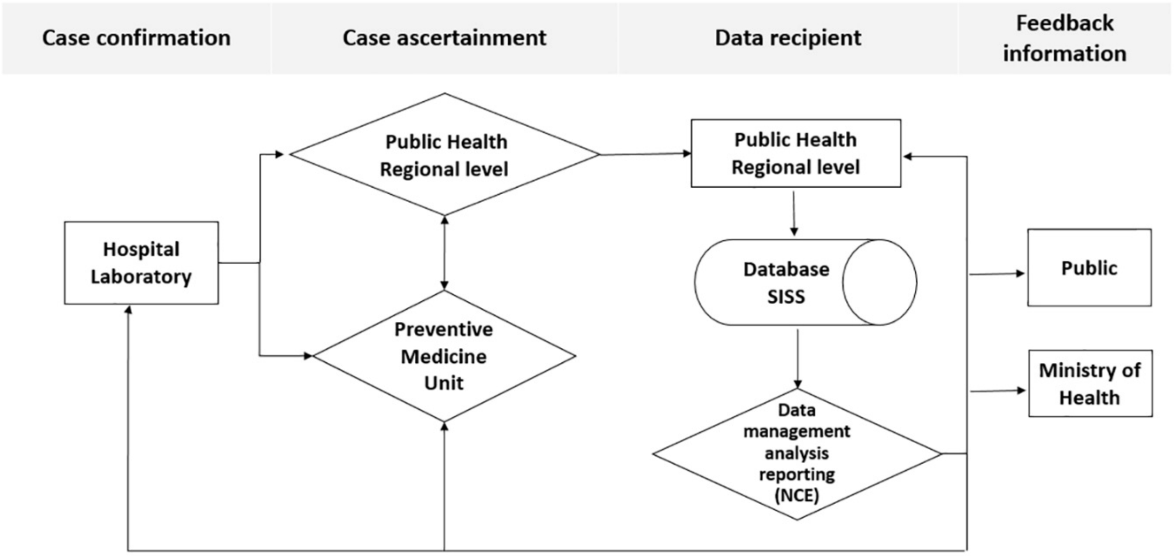

Appendix Figure. Flowchart of Spanish Severe Hospitalized confirmed Influenza cases-patients surveillance system.
